# Impact of university students’ awareness and attitudes on vaccination practices for human papillomavirus, and perception on self-sampling for cervical cancer screening

**DOI:** 10.1186/s40545-022-00471-7

**Published:** 2022-10-29

**Authors:** Mathumalar Loganathan Fahrni, Muhamad Zabidi Azni, Nurhani Syafiqah Mohd Rusdi, Chee-Yan Choo, Khairil Anuar Md Isa, Zaheer-Ud-Din Babar

**Affiliations:** 1 Faculty of Pharmacy, Universiti Teknologi MARA (UiTM) Puncak Alam Campus, Selangor Branch, 42300 Bandar Puncak Alam, Selangor Darul Ehsan Malaysia; 2 Allday Pharmacy Ayer Keroh, 75450 Ayer Keroh Melaka, Malaysia; 3 Faculty of Health Sciences Universiti Teknologi MARA, Selangor Branch, Puncak Alam Campus, 42300 Bandar Puncak Alam, Selangor Darul Ehsan Malaysia; 4 University of Huddersfield, Huddersfield, HD1 3DH West Yorkshire UK

**Keywords:** Vaccination, Primary prevention, Uterine cervical neoplasms, Early detection of cancer, Papillomaviridae, Predictive value of tests, Diagnostic self-sampling

## Abstract

**Background:**

The burden of Human Papilloma Virus (HPV)-associated cancer remains high in developing nations.

**Aims:**

To assess the impact of self-reported awareness and attitudes on vaccination practices, and the perception on self-sampling for cervical cancer screening.

**Methods:**

A 12-month survey using purposive sampling of females attending an urban public university was conducted. SPSS version 25 was used to compare the responses for students enrolled in health vs non-health related programmes.

**Results:**

Of the 290 questionnaires distributed, 240 were returned (response rate = 83%) in approximately equal proportion from the faculties of Health Science and Pharmacy (*n* = 127), and from the Hotel and Tourism, Business Management, and Art and Design (*n* = 113) faculties. About one-third (28.8%) had completed 3 shots, 19.6% received the first shot, 11.4% had scheduled appointments for first shots while 40.2% were both unvaccinated and had not scheduled any appointment. Most (71%) were aware of the HPV vaccines while 50.5% were unaware that HPV vaccines were also available for men. Students enrolled in health-related programmes were 3.2 times more perceptive to the benefits of vaccination particularly in preventing spread to their partners (OR 3.2, 95% CI 1.3–3.41, *p* = 0.006) than their counterparts. A weak-positive correlation was observed between knowledge and vaccination practices (*r* = 0.2, *p* = 0.001). The level of knowledge on HPV and its vaccine was greater for health-related (Mdn = 6.5) than for students of non-health related (Mdn = 1.5) programmes (*U* = 2790*.5*, *p*-value = 0.00). Attitudes towards immunisation were influenced by perceived benefits versus risks for side effects, cost barriers, and influences of primarily their doctors and parents. The study was limited in that relationship statuses were used to estimate sexual history as direct questions were unanswered in the pilot survey.

**Conclusion:**

HPV vaccine uptake for an immunisation-targeted young female population is low despite moderate knowledge levels. It is plausible that the low rates among females enrolled in particularly the non-health programmes were impacted by misperceived vaccine-associated risks, and misconception that testing and vaccination for HPV and cervical cancer were for those married or sexually active. Self-sampling could offer a potential alternative to sampling via pelvic examination, particularly for societies where premarital sex is seen as a taboo.

## Introduction

In 2022, it is 16 years since the vaccine against human papillomavirus (HPV) was first introduced in the United States of America [1]. Yet, HPV remains the most common cause of sexually transmitted diseases in the country. Based on data from 2014 to 2018, approximately 46,143 new cases of HPV-associated cancers were reported in the USA each year, including about 25,719 among women and about 20,424 among men [2]. The Food and Drug Administration (FDA) licensed the bivalent HPV vaccine (HPV2; Cervarix, GlaxoSmithKline) in 2009 and recommended it for routine vaccination in females aged 10 through 25 years. The Advisory Committee on Immunization Practices (ACIP) also recommended vaccination for females aged 13 through 26 years and males aged 13 through 21 years who were not previously vaccinated. Vaccination is also recommended through age 26 years for men who have sex with men and for immunocompromised persons, if not previously vaccinated [3, 4].

In the UK, the HPV vaccine was introduced in 2008 as part of the UK's HPV vaccination programme for those aged 12–13 years. The programme led to a reduction in the incidence of cervical cancer by 87% in women in their 20s in England who were offered the vaccine when they were in school [5]. Vaccination has shown to prevent 90–100% of new high-risk HPV infections among women not infected with HPV at the time of vaccination. Since its introduction, the incidences of 4- and 9-valent type HPV infections have reduced among vaccinated women. The vaccine is also effective at preventing HPV-related anogenital diseases in vaccinated men and reduces the prevalence of oral HPV among young adults [3, 6].

While various strains of HPV exist, formation of genital warts is the most common characteristic. Few notable strains, such as HPV-16 and HPV-18, were frequently associated with the development of cancer. In men, if left untreated, these forms of the virus can lead to cancers of the penis, anus, and the oropharynx. The risks for such cancers were increased particularly among males-sex with males. Among females, in who nearly 60% of associated cancers were seen, there was a pronounced likelihood for untreated infections to develop into precancerous lesions which may metamorphose into full blown cervical cancers [7]. The provision of HPV vaccines was projected to prevent 89% of cervical cancer caused by HPV-16 and HPV-18, and save substantial annual costs for HPV-related morbidities [8].

Together with vaccination, early detection has demonstrated a decline in the incidence of cervical cancer and related deaths, especially among women who received prompt treatment for precancerous lesions and whose prognosis for a 5-year chance of survival was nearly 100% [6]. Nevertheless, national screening programmes across the globe have shown low uptake and pelvic examinations were often viewed as invasive and posed a challenge in itself [6]. A young female population is an integral part of immunisation campaigns and is a target audience for educational programmes. Hence, the aim of this study was to assess female university students’ self-reported awareness and attitudes towards HPV immunisation, and the perception on self-sampling for cervical cancer screening.

## Methods

### Study design and setting

The cross-sectional study was conducted at one of Malaysia’s leading higher learning institutions with one main and 35 satellite campuses. MARA University of Technology’s Puncak Alam campus was purposively selected for inclusion, as the location was feasible and allowed data collection to be conducted systematically.

### Study population and sampling

The study was conducted within a period of 12 months. Data were collected across the campus from July 2017 to June 2018 using structured questionnaires which were self-administered. Participants were chosen using a non-probability sampling technique.

#### Recruitment process

The data were collected by a researcher and assisted by a co-investigator [MAZ and NSMR]. Potential participants were approached outside lecture halls and tutorial rooms. Initially, those potential participants were invited to participate. Then, they were given the participant information sheet and informed consent form containing information on the study’s background, confidentiality aspects, procedures, and risks and benefits associated with the survey. After the participants had signed the informed consent forms and agreed to participate in the study, they were then handed the questionnaire.

The cover letter attached to the questionnaire carried relevant instructions which aided respondents to self-administer. Once completed, the questionnaire was sealable in an envelope, thus ensuring confidentiality and anonymity of the respondents. The researcher then collected the sealed envelopes containing the completed questionnaires. None of the respondents could be traced to the returned questionnaires.

#### Ethical consideration

Approval to conduct this study was obtained from the Research Ethics Committee of the university. Details on the ethical approval are available at the end of this article.

#### Inclusion and exclusion criteria

The respondents were selected based on the following criteria:

Inclusion criteria:

i. Malaysian females,
ii. Age equal or more than 18 years,
iii. University students present on campus.

Exclusion criteria:

i. Respondents who could not understand, read and write in the English language,
ii. Incomplete questionnaires,
iii. Not willing to participate in the study.

#### Sample size calculation

A single proportion formula was used to calculate the target sample size required for the purposive sampling. The formula used was:

$$n=\frac{{z}^{2} \times p(1-p)}{{e}^{2}},$$

where *n* = sample size, *z* = confidence level (*z*-score), *p* = proportion and *e* = margin of error.

Thus, by using the said equation, where *z* = 1.96, *p* = 0.5, and *e* = 0.07, the n was calculated as $n= 196$ respondents. The sample size was increased by 10% to allow for incomplete forms and responses. Hence, the minimum number of respondents required to give the study findings sufficient statistical significance was 216 respondents.

### Survey instrument

The questionnaire used in the study was adapted from previously published questionnaires of studies entitled, “Beliefs and attitudes regarding HPV vaccination among college-age women: an application of the health belief model” [9] and “Knowledge and attitudes regarding the human papillomavirus and HPV vaccine among college students: a gender comparison study” [10].

The questionnaire consisted of three parts:

- Part A—Demographic information about the respondents.
- Part B—Knowledge about the human papilloma virus, vaccines, and self-sampling.
- Part C—Attitudes and perception on HPV and the HPV vaccine.

A pilot study of 15 students whose data were not included in the analysis was done. The necessary changes were then made to the questionnaire. The first part of the questionnaire included respondents’ demographic data and HPV immunisation history. The demographic details of the respondents included age, current year of study, and relationship statuses. Relationship statuses were used to estimate sexual history as direct questions were unanswered in the questionnaire from the pilot study. The HPV immunisation history included information on previous HPV vaccine uptake. The second part of the questionnaire assessed respondents’ knowledge about the virus, HPV vaccine, and self-sampling. The third part of the questionnaire evaluated respondents’ attitudes and perception on the HPV and HPV vaccines. The statements were presented as multiple-choice responses and true or false statements. The responses were assessed on a 5-point Likert scale from strongly agree to strongly disagree. Respondents who answered more than 70% of the questions accurately were considered to have had “good” knowledge, 50–70% accuracy were considered “moderate” while those with fewer than 50% of accurate answers were considered to have had “poor” knowledge. Those who were positive (answered strongly agreed and agreed) to 70% of the statements were considered to have had “good” attitudes and perception, 50–70% were considered “moderate” while respondents with fewer than 50% of positive answers were considered to have had “poor” attitudes and perception.

### Outcome measures

Participants’ socio-demographic information—such as age, relationship statuses (used to extrapolate to sexually active statuses), education level, and family history of cervical cancer were obtained. Data relating to participants’ knowledge, attitudes, and vaccination practices for HPV, HPV testing and self-sampling were also obtained. Knowledge was construed as the respondents’ state of awareness about an HPV infection, its relationship with cervical cancer, asymptomatic nature, mode of transmission and prevention, vaccine availability and accessibility, and testing for HPV including self-sampling. Attitude was construed as the respondents’ perceptions (whether positive or negative) about the disease, and the practice of HPV testing and willingness to self-sample for the test.

### Data analysis

Data collected from the survey were analysed using the Statistical Package for the Social Sciences Statistics (SPSS) Version 25. Mean ± SD or median (interquartile range) was used to express continuous variables. The Chi-square test or Fisher’s exact test was used to compare categorical variables which were expressed as proportions where appropriate. Associations between respondents’ demographic characteristics and the mean ranks of their knowledge on the HPV and vaccination, as well as their attitudes and perception on self-sampling were evaluated using Mann–Whitney, Spearman correlation and logistic regression. All statistical tests were performed at a priori significance level of *p* = 0.05.

## Results

### Demographic characteristics

Of a total 290 questionnaires that were handed out, 240 questionnaires were completely filled and returned—thus, the response rate was 83%. The questionnaires were returned in almost equal proportions from the health-related Faculties of Health Sciences and Pharmacy (*n* = 127), and from the non-health related Faculties of Hotel and Tourism, Business Management, and Art and Design (*n* = 113). The detailed characteristics of the respondents are presented in Table 1.

**Table 1:** Sociodemographic characteristics

Characteristics	*N* (%)
Age (years)	
21	60 (25)
22	131 (54.5)
23–25	49 (20.5)
Undergraduate year of study (year)	
2nd	55 (23)
3rd	154 (64)
4th	31 (13)
Faculty	
Health Sciences	76 (31.6)
Pharmacy	51 (21.3)
Hotel and Tourism	21 (8.8)
Business Management	70 (29.1)
Art and Design	22 (9.2)
Relationship statuses	
In a relationship/ married or intend to get married in the next year	108 (45)
Not in a relationship	132 (55)
Vaccination statuses	
Completed the HPV immunisation (3 shots)	69 (28.8)
First shot only	47 (19.6)
Had not received any shot but had scheduled an appointment with their respective doctor	28 (11.4)
Had not received the HPV vaccine and had not scheduled an appointment with their respective doctor	96 (40.2)

### Vaccination status

**Fig. 1:**
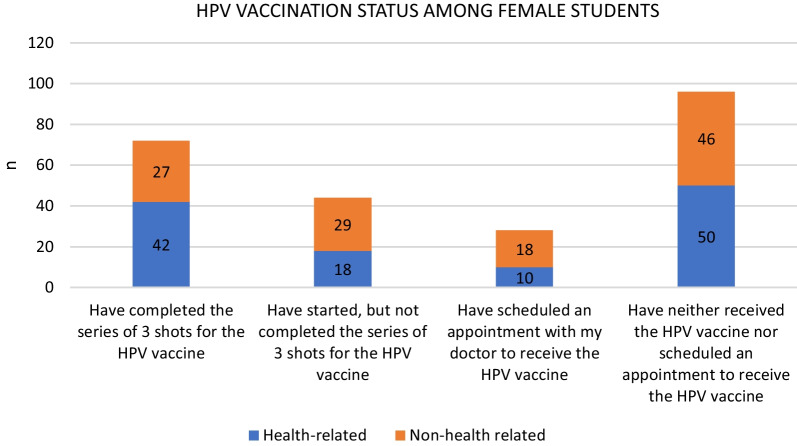
Comparison in vaccination status between health-related and non-health related female students

Approximately one-third of the students (28.8%) had completed their HPV immunisation through a series of 3 shots of the HPV vaccine. Approximately a fifth (19.6%) had received the first shot, 11.4% had scheduled appointments for their first shots, while the remaining 40.2% had neither received the HPV vaccine nor had they scheduled an appointment with their respective doctors for the HPV vaccine (Fig. 1).

### Awareness

As depicted in Table 2, among the female respondents, a majority (87%, *n* = 209) were aware about the Human Papillomavirus even before answering the questionnaire while a minority (7%, *n* = 16) had never heard about the HPV prior to answering the questions. The remaining 6% (*n* = 15) were unsure about whether or not they had heard about the virus. Most females were also aware of the availability of vaccines (71%, *n* = 170) for the prevention of HPV infections, a small percentage (11.5%, 28) were unaware about the HPV vaccine and 17.5% (*n* = 42) indicated that they were unsure about their awareness of the HPV vaccine. Only 9.5% (*n* = 23) were aware that the HPV vaccine was also available for men while most (50.5%, *n* = 121) were unaware of such circumstances and the remainder (40%, *n* = 96) were unsure.

**Table 2:** Awareness of HPV and HPV vaccine

Questions	Yes, N (%)	No, N (%)	Not sure, N (%)
1. Heard of HPV (human papillomavirus) before taking this survey?	209 (87)	16 (7)	15 (6)
2. Heard of the vaccine against HPV (human papillomavirus) (Gardasil^®^, Cervarix^®^) before taking this survey	170 (71)	28 (11.5)	42 (17.5)
3. HPV vaccine is available for men	23 (9.5)	121 (50.5)	96 (40)

#### Sources of HPV and HPV vaccine information

From a multiple choice of eight options, many respondents selected several options and  revealed that a large amount of  HPV and vaccine-related information were sourced from their healthcare providers (47.5%) followed by the internet (44.3%), television or radio (37.9%), school (37.4%), friends (28.3%), newspaper or magazines (23.3%), family (13.7%) and other sources 2.73%.

#### Knowledge on HPV

Table 3 depicts awareness on health-related issues stemming from HPV. From a set of multiple choice questions, n = 182 identified that cervical cancer was linked to HPV. Further analysis revealed that 77% and 74% of the total health and non-health related students concurred with the statement respectively. A few of the respondents (7.5%) inaccurately linked the human immunodeficiency virus to the HPV. Overall, far fewer accurately linked penile cancer to the HPV. Approximately one third (32.5%) successfully associated genital warts to the HPV. Fewer than 17% admitted to not knowing the answers to those statements.

**Table 3:** Health issues related to HPV

Type of students	Cervical cancer is linked to HPV, N (%)	HIV is linked to HPV, N (%)	Penile cancer is linked to HPV, N (%)	Genital warts are linked to HPV, N (%)	Don’t know, N (%)
Health-related	98 (77)	12 (9.4)	10 (7.9)	57 (44.9)	10 (7.9)
Non-health related	84 (74)	6 (5.3)	4 (3.5)	21 (18.6)	30 (26.5)
Total	182 (76)	18 (7.5)	14 (5.8)	78 (32.5)	40 (16.7)

#### Mode of transmission and preventive measures

As presented in Table 4, every respondent in the health-related group was aware that HPV was not transmitted through coughing and sneezing while a minority (9.7%) in the non-health related group was unaware. About half of the respondents knew that the HPV was transmitted through genital skin–skin contact (49%) while 24.5% knew about contact with bodily fluids. As displayed in Table 5, a majority agreed that HPV infections can be prevented by getting vaccinated (68%). Twenty percent agreed on the use of condoms during sexual intercourse while a small proportion (12.5%) had the notion that abstinence was the most effective preventive measure (Table 5). 

Students enrolled in Health-related programmes also stated that HPV vaccines prevented the development of cervical cancer (78%), genital warts (57%), herpes (35%), oral cancer (10%), while 6% did not know the answer. In comparison, for the non-health related group, the percentages of females who agreed with the statements on cervical cancer (60%), genital warts (21%), herpes (10%), and oral cancer (7%) were decreased except for an increase seen in the numbers who did not know the answers (35%).

**Table 4:** Mode of transmission of HPV

Type of student	Coughing and sneezing, N (%)	Genital skin-to-skin contact, N (%)	Contact with bodily fluids (blood, etc.), N (%)	Don’t know, N (%)
Health related	0 (0)	79 (62.2)	25 (19.7)	16 (12.6.)
Non-health related	11 (9.7)	38 (33.6)	34 (30)	37 (32.7)
Total	11 (4.6)	117 (49)	59 (24.5)	53 (22)

**Table 5:** Prevention of HPV infections

Type of student	Prevent HPV infections by practising abstinence, N (%)	Prevent HPV infections by taking antibiotics, N (%)	Prevent HPV infections by using condoms, N (%)	Prevent HPV infections by getting vaccinated, N (%)	Don’t know, N (%)
Health related	17 (13.4)	6 (4.7)	31 (24.4)	100 (78.7)	12 (9.4)
Non-health related	13 (11.5)	16 (14.2)	17 (15)	64 (56.6)	29 (25.7)
Total	30 (12.5)	22 (9.2)	48 (20)	164 (68)	41 (17)

#### Side effects of the HPV vaccine

Many respondents were unaware of the side effects that can arise from the administration of the HPV vaccine. A proportion (39%) did accurately identify that soreness at the site of the injection was the main side effect. A greater proportion of non-health related (54%) respondents versus 34.6% of their counterparts did not know the answer (Table 6).

**Table 6:** Main side effects associated with the administration of the vaccine

Type of student	Vomiting, N (%)	Soreness at the site where the shot is given, N (%)	Headache, N (%)	Joint pain, N (%)	Don’t know, N (%)
Health related	17 (13.4)	47 (37)	5 (3.9)	7 (5.5)	44 (34.6)
Non-health related	7 (6.2)	47 (41.6)	10 (8.8)	11 (9.7)	61 (54)
Total	24 (10)	94 (39)	15 (6)	18 (7.5)	106 (44)

### Attitudes

#### Perceived-benefits of the HPV vaccine

As depicted in Table 7, both health and non-health respondents had positive mindsets that the HPV vaccine was effective in preventing cervical cancer (cumulative percentages of “strongly agreed” and “agreed” were 35.5% versus 33%). Vaccines were perceived as essential in preventing spread to their partners (35% in the former group versus 25% in the latter).

**Table 7:** Perceived-benefits associated with the vaccine

Vaccine effectiveness	Health related (N, %)	Non-health related (N, %)	*P* value
Vaccine is effective in preventing cervical cancer	85 (35.5)	79 (33)	0.124
Vaccine is effective in preventing some types of penile cancer	47 (19.5)	52 (21.5)	0.309
Vaccine is effective in preventing oral cancer	17 (7)	36 (15)	*0.014
Vaccine is effective in preventing the spread of HPV to partners	84 (35)	60 (25)	*0.006

* Statistically significant difference between health and non-health groups (Chi-square, *P* ≤ 0.05)

Many perceived a HPV infection as serious: 24.5% and 22% in the former and latter group respectively believed that, should one contract a HPV infection, it could cause serious harm to their health.

#### Perceived-influences of external sources

Doctors were perceived as the most influential external source, followed by the students’ parents, their partners and then their friends (Table 8). Approximately a third (29%) and 22.5% of those from health and non-health related groups respectively, had the perception that if the people close to them knew about the vaccine from them, they would most likely also get themselves vaccinated against HPV. A minority felt that letting others know that they were getting vaccinated was an embarrassment (5% versus 15.5%).

**Table 8:** Perceived-influences of external sources

External influences	Health-related (N, %)	Non-health related (N, %)	Total (N, %)	*P* value
If my friends knew about the HPV vaccine, they would approve of me getting vaccinated against HPV	48 (20)	40 (16.5)	88 (36.5)	0.513
If my parents knew about the HPV vaccine, they would approve of me getting vaccinated against HPV	53 (22)	46 (19)	99 (41)	0.386
If my partner knew about the HPV vaccine, he/she would approve of me getting vaccinated against HPV	49 (20.5)	46 (19)	95 (39.5)	0.114
If my doctor knew about the HPV vaccine, he/she would approve of me getting vaccinated against HPV	62 (26)	47 (19.5)	109 (45.5)	*0.025
If they knew about the HPV vaccine, most people who are important to me would get themselves vaccinated against HPV if they were at risk	70 (29)	54 (22.5)	124 (51.5)	0.062
If other people knew I received Gardasil, I would be embarrassed	12 (5)	37 (15.5)	49 (20.5)	*0.000

* Statistically significant difference between health and non-health groups (Chi-square, *P* ≤ 0.05)

#### Perceived barrier to vaccination

Approximately one fifth of the students perceived the pricing of the HPV vaccines as expensive (24% health-related versus 23% of non-health related students).

Almost equal numbers in both groups (16% and 17%) thought that the HPV vaccine injection will be painful. A minority, 6.5% versus 14.5%, contemplated vaccination because getting a Gardasil© injection would have been against their belief systems (Table 9).

**Table 9:** Perceived barrier to vaccination

Attitude	Health-related	Non-health related	*P* value
In my opinion, Gardasil is expensive (~ RM900 for the entire series)	58 (24%)	55 (23%)	*0.016
The Gardasil injection will be painful	38 (16%)	41 (17%)	0.887
Getting Gardasil would be against my beliefs	6.5%	14.5%	*0.002

* Statistically significant difference between health and non-health groups (Chi-square, *P* ≤ 0.05)

#### Willingness to self-sample

Although a high percentage had not heard about the Evalyn^®^ brush (62% versus 79%), both groups had positive perceptions and were impressed by the simple self-sampling alternative to using a speculum during pelvic examination. In addition, they were more willing to use self-sampling test kits at home rather than at clinics or health centres.

### Comparison between health-related and non-health related students

Students enrolled in health-related programmes were more perceptive to the benefits of getting vaccinated: in particular, they were 3.2 times more convinced that spread to partners can be prevented by vaccination (OR 3.2, 95% CI 1.3–3.41, *p* = 0.006) than their counterparts. In addition, there were significant associations in the numbers between the two groups who thought that vaccination prevented oral cancer (*p* = 0.014), who were convinced that their doctors would approve of their decision to get vaccinated (*p* = 0.025), who would be embarrassed if others knew they got vaccinated against the HPV (*p* = 0.000), who thought that the vaccines were expensive (*p* = 0.016), and whose beliefs were against vaccination (*p* = 0.002). The health group fared better than the non-health group. A weak-positive correlation was observed between knowledge and vaccination practices (*r* = 0.2, *p* = 0.001). The level of knowledge on the HPV and its vaccines was greater for health-related (Mdn = 6.5) than for non-health related female students (*Mdn* = 1.5), *U* = 2790.5, *p*-value = 0.00).

## Discussion

### Vaccination practices

The method of hand-delivering the questionnaires generated good survey response rates among the population sampled [11]. Our study had the added advantage of having obtained data from students enrolled in faculties which were both health- and non-health related. This allowed a better reflection on the dispersion of awareness and attitudes on HPV and its vaccination, and perception on self-sampling to screen for HPV and cervical cancer. Similar to the implications of this study, a study in Morocco found that non-health related students were less willing than health-related students to get vaccinated [12].

The broad coverage yet low vaccine uptake evident in our study where a cumulative 48.4% were at least vaccinated once was also evident in the USA. Adolescent HPV vaccination rates remain suboptimal and those rates are even lower among older-aged college students, with 18- to 21-year-old female and male college students more likely to be vaccinated at least once than the 22- to 26-year-old female and male college students [13]. Creating an awareness and engaging all target population is ultimately the goal in cultivating better preventive practices. It is indeed pertinent for students studying health-related courses to provide satisfactory and accurate counselling to the public when they become healthcare professionals [14].

### Awareness, attitudes and perception on screening and vaccination

The study demonstrated that Malaysia being a relatively advanced and middle-income country still faced challenges related to consumers’ awareness on vaccination. While 71% knew about the HPV vaccines, one half did not know that the vaccines were also available for men. Similarly, in sub-Saharan Africa, a study which focused primarily on assessing young people’s knowledge, attitude and practices concerning cervical cancer reported that participants knew little about the HPV. The study on attendees of high school and universities reported that less than half (47%) of the participants knew about the HPV transmission and prevention [15]. More awareness among students of Asian origin is needed on sensitive topics such as women’s and men’s health.

Although cervical cancers are mostly preventable with screening and early detection, other HPV-associated cancers like anal, vaginal, oropharyngeal, vulvar, and penile are yet to have established screening guidelines [1]. This highlights the need for vaccination against HPV-associated infections. Awareness on both cervical cancer as well as HPV screening can greatly increase the uptake of vaccination. Moreover, with more infectious diseases emerging due to deforestation and ecological neglect, vaccination will be key for their containment [16, 17].

### Highest risk group for cervical cancer

In Malaysia, cervical cancer, is still the second most frequently occurring cancer (after breast cancer) among women aged 15–44 years [8]. The Ministry of Health, through its inter-agency and multi-sectoral collaborations, implemented the HPV school-based immunisation programme for 13-year-old girls or first year secondary students in 2010 and fully covered its cost. Since then, the vaccines, Gardasil (was available for purchase in 2006) and Cervarix, were registered and available for out-of-pocket paying individuals, and the target population was those aged 9–40 years [18]. Yet, as demonstrated in our study, the numbers of those fully immunised against the HPV was a mere 28.8%. The low uptake rate for an immunisation-targeted population is of concern.

In a recent study in Iraq, where molecular analysis using polymerase chain reaction was performed on cervical samples of women aged 32–78, the incidence of HPV-16 infection was higher in younger women, while infection rates declined in older women [19]. In several low-income countries in Africa and Asia, HPV infections have also been most prevalent among younger women of ages 16–22 years [6]. The challenge in low-income to middle countries however, depended on striking a balance between the associated benefits of testing (i.e., sensitivity and positive predictive values [PPV] for high-grade cervical intraepithelial neoplasia) and cost barriers (i.e., colposcopy referral and false-positive rate).

### Self-sampling can overcome barriers to testing

Prior to the introduction of vaccines in 2010 in Malaysia, cytology-based screening was the primary preventive measure for cervical cancer. Screening services using the Papanicolaou smear (Pap smear) was initially aimed at post-partum mothers in family planning clinics. In 1995, the services were also offered to women aged 20–65 years [20]. Despite promotional campaigns on screening services nationwide, the uptake rates were only 26% in 1996 and 43.7% in 2006 [21].

The burden of cervical cancer in the Asia pacific region is moderately high due to the limitations conferred by cytology-based screening. Lower income countries might not get the best preventive healthcare or equitable access to the vaccines or diagnostic tests in comparison to the more advanced and higher income countries [22]. The cost for the recommended doses of the full course for HPV vaccines was approximately $360 USD or MYR 1200, which many families with lower socio-economic statuses could not afford [5]. In addition, parental or legal guardian consent is required for young adults aged below 18 years.

Self-sampling for HPV testing can be a cost-effective alternative to clinic-based cervical screening. For detecting cervical intraepithelial neoplasia grade 2 or more (CIN2 +), repeated HPV self-sampling in comparison with cytology-based (pap smear) screening was a cost-effective alternative. The study involved a cost-effectiveness analysis of participants who were randomised to either undergo repeated HPV self-sampling of vaginal fluid (*n* = 17,997) or to undergo midwife-collected Pap smears for cytological testing (*n* = 18,393). A higher number of women screened at a lower cost in the self-sampled versus midwife-collected Pap smears group (€ 229,446 vs. € 782,772) [23].

### Strengths and limitations

The study has an added strength of having surveyed end-users’ perception on self-sampling as an alternative technique to sampling via pelvic examination. The study’s recruitment process however, had its disadvantages in that, sampling did not account for the proportion of students enrolled in each faculty. Nevertheless, the comparable number of samples in  both groups allowed for a comparison with statistical significance. In addition, the study was limited in that relationship statuses were used to estimate sexual history as direct questions were unanswered in the pilot survey.

## Conclusion

Despite moderate knowledge levels, the overall vaccination uptake for the human papillomavirus is low for a young female population. In comparison with students enrolled in health-related programmes, the non-health related students had lower knowledge levels. However, the attitudes and perception on self-sampling for screening were comparable for the two groups. Knowledge levels of students correlated with vaccination practices and this was evident in the more encouraging HPV vaccine uptake among health-related students when compared to their counterparts. Factors which plausibly impacted vaccination practices were misperceived risks associated with the vaccines and the misconception that, testing for HPV, cervical cancer screening and vaccination, were for the married or sexually active population. Finally, self-sampling could offer a potential alternative technique to sampling via pelvic examination. This would  address cultural barriers and in particular, benefit the societies where premarital sex is seen as a taboo.

## Data Availability

The data that support the findings of this study are available in the additional material section of this article.
